# Synthesis, Bottom up Assembly and Thermoelectric Properties of Sb-Doped PbS Nanocrystal Building Blocks

**DOI:** 10.3390/ma14040853

**Published:** 2021-02-10

**Authors:** Doris Cadavid, Kaya Wei, Yu Liu, Yu Zhang, Mengyao Li, Aziz Genç, Taisiia Berestok, Maria Ibáñez, Alexey Shavel, George S. Nolas, Andreu Cabot

**Affiliations:** 1 Departamento de Física, Universidad Nacional de Colombia, Ciudad Universitaria, Bogotá 111321, Colombia; 2 Department of Physics, University of South Florida, Tampa, FL 33620, USA; kayawei@mail.usf.edu; 3 Catalonia Institute for Energy Research-IREC, Sant Adrià de Besòs, 08930 Barcelona, Spain; liuyu92525@gmail.com (Y.L.); tingchan99@gmail.com (Y.Z.); limengyaorz@gmail.com (M.L.); taisiia.berestok@gmail.com (T.B.); al.shavel@gmail.com (A.S.); 4 Am Campus 1, Institute of Science and Technology Austria, 3400 Klosterneuburg, Austria; mibanez@ist.ac.at; 5 Department of Materials Science and Engineering, Faculty of Engineering, Izmir Institute of Technology, Urla, İzmir 35430, Turkey; azizgenc@gmail.com; 6 ICREA (Institució Catalana de Recerca i Estudis Avançats), Pg. Lluís Companys 23, 08010 Barcelona, Spain

**Keywords:** nanocrystals, thermoelectrics, bottom-up engineering, doping, chalcogenides, lead sulfide

## Abstract

The precise engineering of thermoelectric materials using nanocrystals as their building blocks has proven to be an excellent strategy to increase energy conversion efficiency. Here we present a synthetic route to produce Sb-doped PbS colloidal nanoparticles. These nanoparticles are then consolidated into nanocrystalline PbS:Sb using spark plasma sintering. We demonstrate that the introduction of Sb significantly influences the size, geometry, crystal lattice and especially the carrier concentration of PbS. The increase of charge carrier concentration achieved with the introduction of Sb translates into an increase of the electrical and thermal conductivities and a decrease of the Seebeck coefficient. Overall, PbS:Sb nanomaterial were characterized by two-fold higher thermoelectric figures of merit than undoped PbS.

## 1. Introduction

To exploit the full potential of bottom-up strategies to produce functional nanomaterials, it is necessary to develop strategies to control the charge carrier concentration [1,2]. However, introducing controlled amounts of electronic dopants into colloidal nanocrystals (NCs) has been often a main challenge. A particular application, where such electronic doping is key and where the use of nanomaterials presents a clear advantage, is thermoelectricity [3,4,5,6]. All relevant thermoelectric properties, electrical conductivity (*σ*), Seebeck coefficient (*S*) and thermal conductivity (*κ*), strongly depend on charge carrier concentration. While *σ* increases with charge carrier concentration, thus improving the thermoelectric figure of merit *ZT* = *σS*^2^/*κ*, *S* decreases and the electronic contribution to *κ* increases, both having a detrimental effect on *ZT* [7,8]. Thus, a precise adjustment of the carrier concentration is necessary to optimize the material’s thermoelectric properties.

The research on thermoelectric materials typically aims to identify good candidate materials and optimize their properties to maximize *ZT*. Since the *ZT* of a material peaks at an optimum carrier concentration, once identified a potential candidate, the strategy to follow is to optimize its charge carrier concentration. At the same time, one needs to decouple as much as possible the three transport parameters, *σ*, *S* and *κ*, to maximize the thermoelectric efficiency [9,10].

Nanostructuring has resulted in high-performance thermoelectric materials, characterized by reduced *κ* due to phonon scattering at grain boundaries [3,11,12,13,14]. Nanomaterials may also display enhanced *S* when correctly designed and precisely engineered. In this scenario, processing of thermoelectric nanomaterials by bottom-up assembly of colloidal NCs is particularly interesting [5,14,15,16,17,18,19,20,21]. Colloidal NCs with controlled size, shape, crystallographic phase and composition are ideal building blocks to produce nanostructured materials with well-tuned parameters [15,16,22,23,24,25,26,27,28]. From a fundamental point of view, these well-controlled systems can be used to gain further understanding of the mechanisms and processes underlying the thermoelectric effect. From an application point of view, such well-controlled systems produced by solution-processing technologies may hold the key to cost-effective thermoelectric devices in a wide range of applications [29].

To produce optimally doped NCs, it is crucial to control their surface chemistry. There is a large number of atoms at the NC surface, which may have dangling bonds that act as traps or electron donors. These dangling bonds may be passivated by organic or inorganic ligands that can be introduced during the synthesis or by ligand exchange, but that may also be adsorbed from the environment, as in the case of oxygen or hydroxyl molecules [30,31,32,33].

Due to its relative simplicity and high performance, one particularly interesting thermoelectric material is PbS. N-type PbS, doped with PbCl_2_ and mixed with a small fraction of a second nano-precipitated phase such as PbTe [34], PbSe [35], Sb_2_S_3_ or Bi_2_S_3_ [36] has reached *ZT* values up to 0.8 at 770 K, 1.3 at 900 K, 0.79 at 723 K and 1.1 at 923 K, respectively. P-type PbS doped with Na and mixed with SrS [37] or CdS [38] secondary phases has reached *ZT* values of up to 1.2 and 1.3 at 923 K, respectively. Regarding the bottom-up approach, Cl-doped PbTe_x_Se_1−x_@PbS core-shell NCs reached thermoelectric figures of merit of up to 0.94 at 700 K [32], and PbS-Ag nanocomposites produced by strategic engineering of nano-scale building blocks and interfaces using PbS NCs resulted in thermoelectric figures of merit of up to 1.7 at 850 K [13]. In general, this last strategy proved successful in introducing metals, such as Cu or Sn, into the materials with reported figures of merit of 0.86 and 0.88 at 855 K [39]. Besides, in the lead chalcogenide family, PbTe with a co-doping of silver and antimony or bismuth, also present higher thermoelectric efficiency due to the inhomogeneities resulting from Ag or Sb segregation [40,41].

In this work, we demonstrate that Sb-doping in colloidal PbS NCs can be employed to tune the carrier concentration of bottom-up processed nanocrystalline materials. We detail here the synthesis protocol to produce the Sb-doped PbS NCs and the results from characterizing the nanomaterial resulting from the spark plasma sintering (SPS) of the NCs at 400 °C. We chose Sb as n-type donor since Sb^3+^ substitutes Pb^2+^ in the case of PbTe [42] resulting in n-type doping [43].

## 2. Materials and Methods

### 2.1. Materials

Lead (II) oxide (PbO, 99.9%), elemental sulfur (99.998%), antimony (III) acetate (99.99%), oleic acid (OAc, tech. 90%), 1-octadecene (ODE, 90%), oleylamine (OAm, tech. 70%), sodium sulfide nonahydrate (NaS·9H_2_O 99.99%), and ethylene glycol (EG, 99.5%) were purchased from Aldrich (St. Louis, MO, USA). Chloroform, hexane, and ethanol were of analytical grade and obtained from various sources. All chemicals were used as received, without further purification. All syntheses were carried out using a vacuum/dry argon gas Schlenk line and argon glovebox for storing and handling air- and moisture-sensitive chemicals.

### 2.2. Synthesis of PbS Nanocrystals

PbS NCs were prepared following a reported procedure [32]. In a typical synthesis, 4.46 g of lead oxide (20 mmol) and 50 mL of OAc (0.159 mol) were mixed with 100 mL of ODE (0.312 mol). This mixture was heated to 130 °C and maintained at this temperature for 1 h under vacuum to form the lead oleate complex. This precursor solution was then flushed with Ar and heated to 210 °C. At this temperature, a sulfur precursor, prepared by dissolving 0.64 g of elemental sulfur (20 mmol) in 20 mL of distilled OAm (0.061 mol), was rapidly injected. Sb-doped PbS NCs were obtained by introducing different quantities (from 2 mmol, 3 mmol and 5 mmol) of Sb (III) acetate in the precursor solution. The reaction mixture was maintained between 195 °C and 210 °C for 5 min and then quickly cooled down to room temperature using a water bath. PbS NCs were washed by multiple precipitation/re-dispersion steps using ethanol as a non-solvent and hexane as solvent.

### 2.3. Removal of the Capping Ligands

In a typical procedure, a stock 0.1 M solution of Na_2_S·9H_2_O in EG was prepared, and as-synthesized PbS NCs (300 mg) were dispersed in 10 mL of chloroform. Ligand exchange was performed by adding 5 mL of a Na_2_S·9H_2_O solution to the NCs in CHCl_3_. After stirring for 30 min under Ar, the solution was precipitated at 3000 rpm. During the stirring, NCs were displaced from chloroform to EG solution. NCs were then thoroughly purified using chloroform to remove the remaining organic species. Finally, the PbS NCs were precipitated, dried and stored in a glove box until posterior use.

### 2.4. Structural and Chemical Characterization of PbS and Sb-Doped PbS NCs

X-ray power diffraction (XRD) analyses were carried out on an AXS D8 Advance X-ray diffractometer (Bruker, Rosenheim, Germany) with Cu Kα1 radiation (λ = 1.5406 Å). The size and shape of the nanoparticles were examined by transmission electron microscopy (TEM) (TEM Libra 120, Zeiss, Oberkochen, Germany) using a Libra 120 (Zeiss, Oberkochen, Germany) operating at 120 keV accelerating voltage and high resolution TEM (HRTEM), high-angle annular dark-field imaging (HAADF-STEM) (Jeol, Tokyo, Japan) and electron energy-loss spectroscopy (EELS) (Zeiss, Oberkochen, Germany) in a Jeol 2010F (Jeol, Tokyo, Japan ) field emission gun microscope operated at 200 kV. Scanning electron microscopy (SEM) (Jeol, Tokyo, Japan) was performed using a Zeiss Auriga microscope (Zeiss, Oberkochen, Germany) with an energy dispersive X-ray spectroscopy (EDX) detector to study the material’s composition.

### 2.5. Consolidation and Thermoelectric Characterization of PbS and Sb-Doped PbS NCs

Nanoparticles were consolidated into dense nanomaterials using spark plasma sintering (SPS) in a commercial instrument (Version: 10–3, Thermal Technology LLC Inc, Santa Rosa, CA, USA). The sintering of PbS and Sb-doped PbS NCs were carried out at 60 MPa and 400 °C for 10 min, resulting in pellets with densities of 90% and 94% of the theoretical value, respectively. The thermoelectric properties of the nanocrystalline materials were characterized by using a custom design radiation-shielded vacuum probe. Temperature dependent four-probe resistivity (ρ = 1/*σ*, *S* (gradient sweep method), and steady-state *κ* were measurements from 12 to 300 K with uncertainties of 4, 6, and 8%, respectively [44,45]. The samples were cut by a wire saw into parallelepipeds of 2 × 2 × 5 mm^3^ in order to conduct these measurements. The Hall measurements were conducted in a four-probe configuration (on 0.5 × 2 × 5 mm^3^ samples cut by a wire saw) using an electromagnet that generated a magnetic field up to 1.2 T. Current was passed through the specimen while the field was varied between 0.1 T and 1 T in increments of 0.1 T with an inversion of the field to eliminated voltage probe misalignment effects.

## 3. Results and Discussion

**Figure 1:**
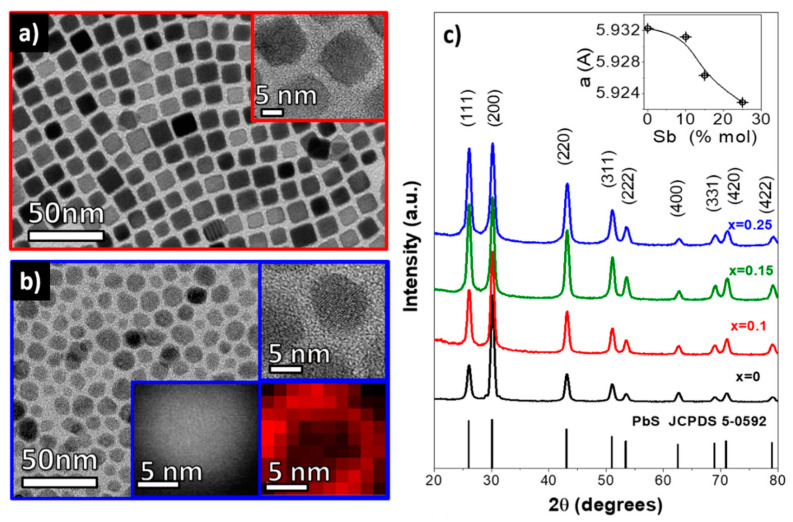
TEM and HRTEM (insets) micrographs of Pb_1−x_Sb_x_S for x = 0 (**a**) and x = 0.15 (**b**) NCs. A HAADF-STEM image and an EELS elemental map with the distribution of Sb within a single PbS nanoparticle are also shown as insets of (**b**). The EELS map is taken by extracting the signal from the M_4,5_ edge of Sb which starts at 528 eV. (**c**) XRD patterns of the Pb_1−x_Sb_x_S NCs obtained with different nominal dopant concentrations of x = 0, 0.1, 0.15 and 0.25.

**Figure 2:**
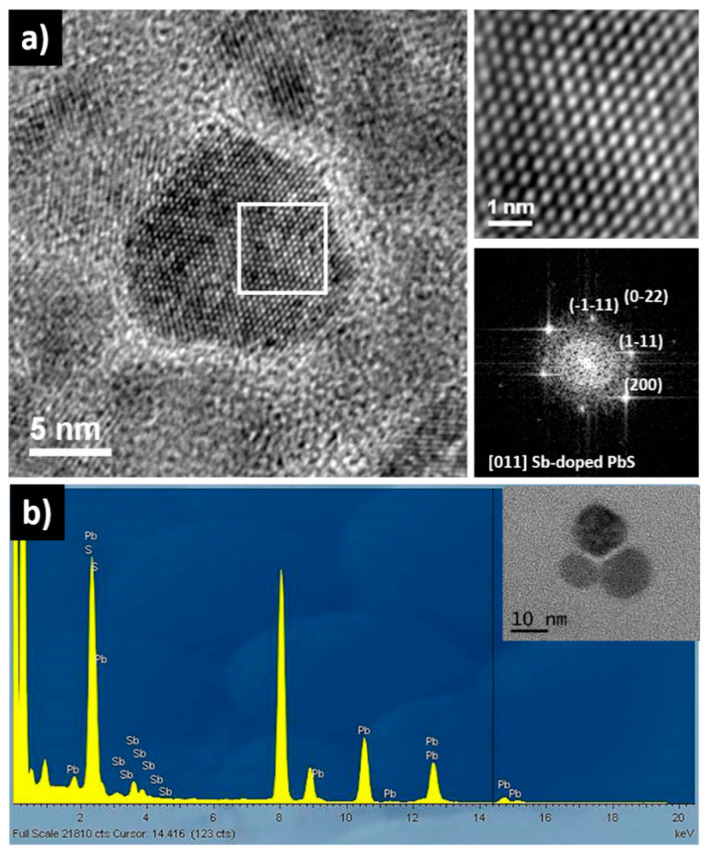
(**a**) HRTEM micrographs of Pb_1−x_Sb_x_S NCs for x = 0.15, the crystal structure could be indexed as the galena PbS with face-centered cubic (fcc) symmetry belonging to Fm3m space group. The Pb_1−x_Sb_x_S was zone axis [011]. (**b**) EDS spectrum of the same Pb_1−x_Sb_x_S NCs showing the presence of Sb. EDS quantified the Sb composition at x = 0.10. Inset shows a TEM image of the NCs.

Figure 1a shows a representative TEM micrograph of the PbS NCs obtained by the reaction of lead oleate with sulfur in the presence of OAm and OAc, following the above-detailed procedure. PbS NCs were highly monodisperse and displayed a cubic morphology. Their size could be controlled in the range from 8 nm to 12 nm by changing the reaction temperature from 130 °C to 210 °C. Their crystal structure was identified as cubic galena (Fm-3m, JCPDS 5-0592) by means of HRTEM (Figure 1a inset) and XRD (Figure 1c). The introduction of Sb ions in the precursor solution clearly influenced the particle morphology. The presence of Sb resulted in irregular shaped Pb_1−x_Sb_x_S NCs having broader size distributions (Figure 1b). The PbS crystallographic phase remained unchanged as observed from HRTEM (Figure 2a) and XRD analyses (Figure 1c). However, the cubic crystal lattice was noticeably modified by the presence of Sb. It was observed that Sb incorporation affected the relative XRD peak intensity and the lattice parameter (Figure 1c inset). Observed differences are related to both the loss of the cubic morphology of the NCs, and to a strain within the formed Pb_1−x_Sb_x_S NCs. No secondary phases were identified in our XRD data even with the specimen prepared at the highest concentration of Sb precursor.

EELS analysis of several Pb_1−x_Sb_x_S NCs did not show a significant change in Sb composition from particle to particle. However, EELS analysis also showed that Sb was not homogeneously distributed within each Pb_1−x_Sb_x_S NC but a radial gradient of Sb was observed, with the Sb concentration higher in the outermost layers of the NC than in the center (Figure 1b inset). In terms of composition, ICP and EDX analysis (Figure 2b), showed the Sb content of the NCs Pb_1−x_Sb_x_S (with x = 0.15 nominal) to be close to x = 0.10.

PbS and Pb_1−x_Sb_x_S NCs were used as building blocks to produce PbS and Pb_1−x_Sb_x_S polycrystalline materials with nano-scale grains. Before NCs consolidation into polycrystalline bulk materials, with the NCs still in solution, the surface ligands used to control the colloidal NCs solubility and growth during the synthesis were displaced using sodium sulfide. This step is considered to be fundamental to achieve high thermoelectric performances. As has been extensively demonstrated in previous works, the exchange of surface ligands in the surface of colloidal NCs affects the carrier mobility of the consolidated nanomaterials [25,46,47]. The removal of OAm/OAc by a Na_2_S·9H_2_O solution improves the NCs interaction and reduces the amount of carbon in the final nanomaterial, which results in an increase of the charge carrier mobility. At the same time, it is well known, that ligands modify the NC surface chemistry [48]. In the particular case of OAc ligands, the annealing of NC-OAc samples usually results in an extensive surface oxidation that can be prevented by the displacement of OAc before sintering.

**Figure 3:**
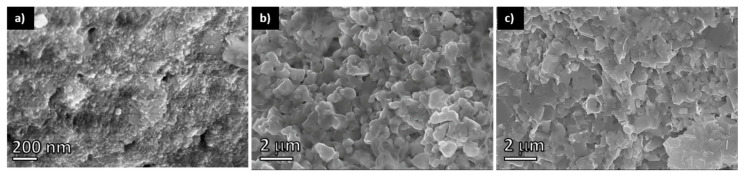
SEM micrographs of: (**a**) PbS nanopowder after ligand exchange; (**b**) PbS consolidated pellet; and (**c**) Pb_0.9_Sb_0.1_S consolidated pellet.

The final NCs were purified from residual organic molecules using chloroform and dried. The resulting nanopowders were consolidated into 12 mm diameter and 2 mm thick disk-shaped pellets using SPS at 400 °C and 60 MPa. The obtained pellets had densities between 90% and 94%. Figure 3 shows SEM micrographs of the consolidated PbS and Pb_1−x_Sb_x_S polycrystalline materials. The crystal size domains increased by a factor of 10 during the SPS process.

**Figure 4:**
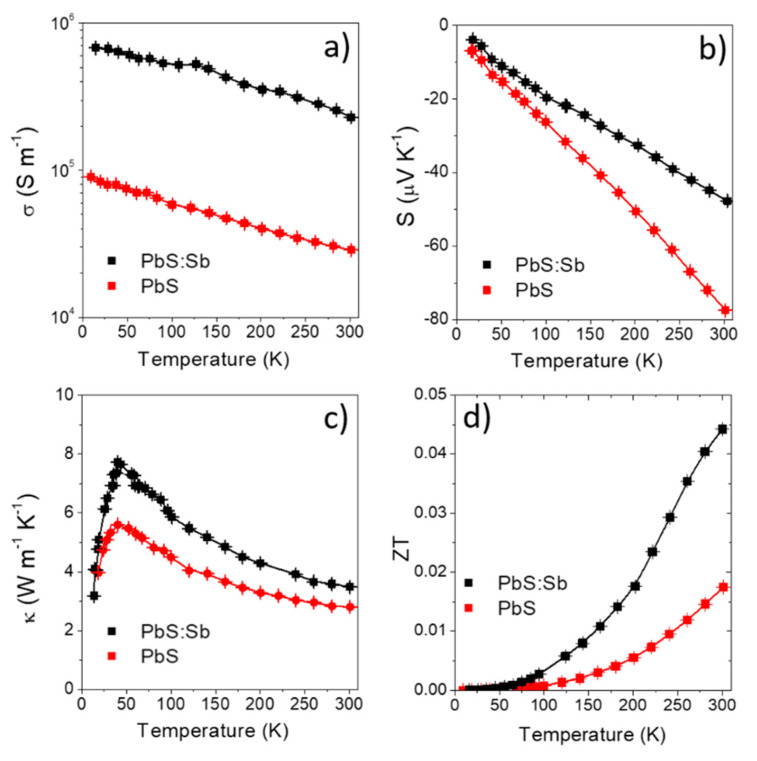
(**a**) Electrical conductivity, (**b**) Seebeck coefficient, (**c**) thermal conductivity and (**d**) thermoelectric figure of merit of PbS (red) and Pb_0.9_Sb_0.1_S (black) nanomaterials.

Figure 4 shows the temperature dependence of *σ*, *S*, *κ* and *ZT* of PbS and Pb_0.9_Sb_0.1_S polycrystalline materials. As expected, *σ* notably increased with Sb doping. Overall, almost a one order-of-magnitude increase in *σ* was observed with doping. On the other hand, a slight relative decrease of *S* and a moderate increase of *κ*, by a 20% and a 30% at 300 K, respectively, were also obtained. S decreases monotonically with the temperature and is negative for both materials. Its absolute value decrease with the Sb doping, due to the increase of the charge carrier concentration. Regarding *κ*, it is important to analyze the behavior of its two contributions, the phononic or lattice thermal conductivity, $\kappa_{L}$, and the electronic thermal conductivity, $\kappa_{e}$. According to the Wiedermann–Franz law, $\kappa_{e}$ increases due to the increase of *σ* for the Sb doped sample, as compared to the undoped sample. Additionally, the curves of κ present a peak around 50 K. This is because heat conduction in lightly/moderately doped semiconductor crystals is dominated by the lattice thermal conductivity contribution $\kappa_{L}$. At low temperatures (T < $\theta_{D}$) the mean free phonon path becomes large and the phonon movement is governed by point defects or boundary scattering, where $\kappa \sim T^{-1},$ whereas, at high temperatures the phonon scattering comes from umklapp processes that lead to a reduction in the mean free phonon path, and $\kappa \sim T^{3}$. Therefore, the peak observed in the trend of κ vs. T curve, Figure 4c, indicates the change in the scattering process between point defect/boundary scattering and umklapp scattering [8,49,50,51].

The large increase of *σ* obtained in the doped sample compensated for the decrease of S and the increase of *κ*, resulting in higher power factors (PF = *σS*^2^) and a two-fold increase in *ZT*. Hall measurements indicated that electrons are the majority carriers for both materials, with room temperature carrier concentrations of 6 × 10^19^ cm^−3^ and 9 × 10^19^ cm^−3^ for PbS and Pb_0.9_Sb_0.1_S, respectively (Table 1). As observed in PbTe:Sb, we assume Sb^3+^ substitutes Pb^2+^ ions providing one extra electron to the PbS conduction band. This n-type doping effect is consistent with the negative value of the Seebeck coefficients and the measured Hall charge carrier concentrations. The carrier concentration of polycrystalline PbS was relatively high, presumably related to an off-stoichiometric composition, PbS was Pb-rich, originated during either the ligand exchange or the consolidation process. This off-stoichiometry is difficult to control, which makes difficult the required tuning of the charge carrier concentration. Our work clearly indicates that it is more feasible to adjust the carrier concentration of bulk polycrystalline materials by doping PbS NCs with Sb.

Finally, Table 2 shows the state-of-the-art values of *ZT* for lead chalcogenide compounds doped with different elements for reference.

## 4. Conclusions

Sb-doped PbS NCs were produced by a novel colloidal synthesis protocol, with Sb radially distributed within the PbS NCs. Sb significantly modified the size, geometry and crystal lattice of PbS NCs. Bulk polycrystalline PbS with nano-scale grains were obtained from the assembly and SPS consolidation of the NCs. Doping of the PbS NCs with Sb allowed for a further increase in the carrier concentration of the consolidated polycrystalline material, resulting in a higher *σ* and a two-fold increase in *ZT* as compared with un-doped PbS.

## Figures and Tables

**Table 1:** Charge carrier concentration and mobility of PbS and Pb_0.9_S_0.1_bS, according to hall measurements.

	Properties	Charge Carrier Concentration cm^−3^	Charge Carrier Mobility cm^2^/V s
Sample			
PbS		6 × 10^19^	30
Pb_0.9_Sb_0.1_S		9 × 10^19^	154

**Table 2:** State of the art of *ZT* values for lead chalcogenides compounds doped or mixed with different elements.

System	*σ* (S m^−1^ × 10^4^)	*S* (µV K^−1^)	*κ* (W m^−1^ K^−1^)	*ZT* (300K)	Ref.
PbS + Sb	23	−48	3.48	0.045	This Work
PbS + 4.6% Ag	8	−50	1.62	0.037	[13]
PbTe_0.1_Se_0.4_S_0.5_-Cl	1	−75	1.25	0.013	[32]
(PbSe)_0.88_(PbS)_0.12_ + 0.3% PbCl_2_	30	−75	2.9	0.17	[35]
Pb_0.98_Na_0.02_S	12.6	82	2.3	0.11	[37]
Pb_0.975_Na_0.025_S + 3.0% SrS	7.5	80	1.6	0.09	[38]
PbS + 4.9% Sn	3.8	−75	1.8	0.035	[39]

## Data Availability

The data presented in this study are available within the article.
